# Field-ready biomarkers for quantifying dehydration: applications for mountain and high-altitude warfighters

**DOI:** 10.1080/15502783.2025.2550166

**Published:** 2025-08-26

**Authors:** S. Kyle Travis, Antonella Schwarz

**Affiliations:** a Liberty University, School of Health Sciences, Department of Allied Health Professions, Lynchburg, VA, USA; b Science, Medicine, Innovation & Technology (SMIT) Laboratory, nøsisX, Elizabethton, TN, USA; c Barry University, Department of Sport and Exercise Sciences, Miami Shores, FL, USA

**Keywords:** Tactical, hydration, noninvasive, fluid loss

## Abstract

**Background:**

Dehydration is a critical threat to performance and safety in tactical settings, particularly during prolonged activity in heat or altitude while wearing heavy gear. Sweat-prone regions like the chest, back, and lower legs, which are areas typically covered by packs, armor, and boots, may experience accelerated fluid loss. This study evaluated whether noninvasive salivary biomarkers and regional skin temperatures could serve as practical indicators of hydration status.

**Methods:**

Ten males (25.5 ± 3.5 yrs, 179.9 ± 7.2 cm, 78.1 ± 8.5 kg) completed a 2-hour treadmill run without fluid intake. Saliva and skin temperature measurements were collected every 15 minutes (8 timepoints). Biomarkers (osmolality, chloride, and cortisol) and regional skin temperatures (chest, back, lower leg) were analyzed relative to dehydration. Total body water (TBW) loss and percent dehydration were used as outcomes. Bayesian Pearson correlations (r) and Bayes Factors (BF_10_) assessed associations, with magnitude and evidence strength classified using Hopkins and Jeffreys scales. Bayesian multiple regression models (R^2^) evaluated predictive contributions of each variable, with model outputs including Bayes factor inclusion scores (BF_incl_) and standardized beta coefficients (ß). Analyses were performed in JASP (v0.19.3).

**Results:**

Salivary osmolality (BF_10_ = 58.00; *r* = –0.142) and chloride (BF_10_ = 53.25; *r* = –0.010) were supported by very strong evidence as predictors of total body water (TBW) loss, though both exhibited small, inverse associations with TBW loss. Cortisol (BF_10_ = 23.55; *r* = 0.311) exhibited strong evidence and a moderate positive correlation, increasing as TBW loss increased. Chest temperature demonstrated the strongest predictive contribution in the regression model (BF_incl_  = 1874.60; ß = –0.167), followed by lower leg (BF_incl_  = 45.82; ß = –0.153) and back (BF_incl_  = 34.81; ß = –0.145). Negative beta coefficients reflected positive associations between rising surface temperatures and greater dehydration. Fluid-based models explained up to 70.6% of TBW loss variance (R^2^ = 0.706), outperforming temperature-only models (R^2^ = 0.451). When models were replicated using percent dehydration as the outcome, results remained consistent; chest temperature remained the most consistently included thermal predictor (BF_10_ = 7.34), though the strength of its association was small.

**Conclusions:**

Salivary osmolality, chloride, and cortisol, along with chest, back, and lower leg temperature, show strong promise as noninvasive, field-ready indicators of dehydration. These findings support the use of portable thermal sensors and saliva-based tools to help tactical personnel monitor hydration status during sustained operations in austere environments.

